# Supplementary figures and images for: An atlas of robust microbiome associations with phenotypic traits based on large-scale cohorts from two continents
Source: PLoS One. 2022 Mar 24;17(3):e0265756. doi: 10.1371/journal.pone.0265756 (PMC8947124; doi:10.1371/journal.pone.0265756)

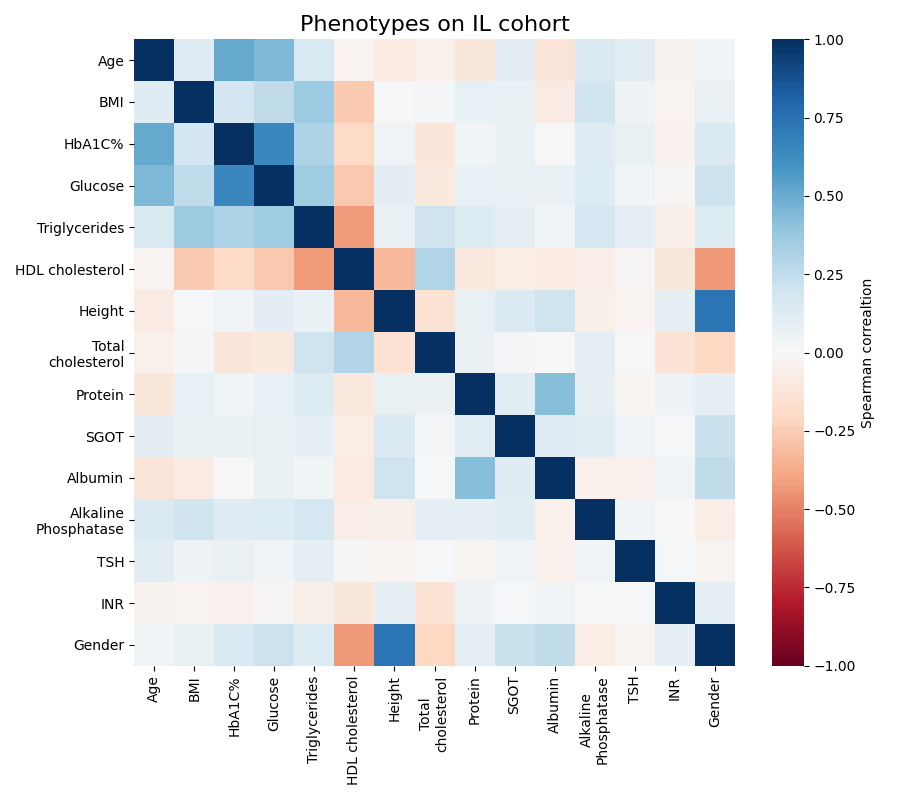

Supplement: S1 Fig — Spearman correlation of the different phenotypes, on the Train-IL cohort. (TIFF) [file pone.0265756.s001.tiff]

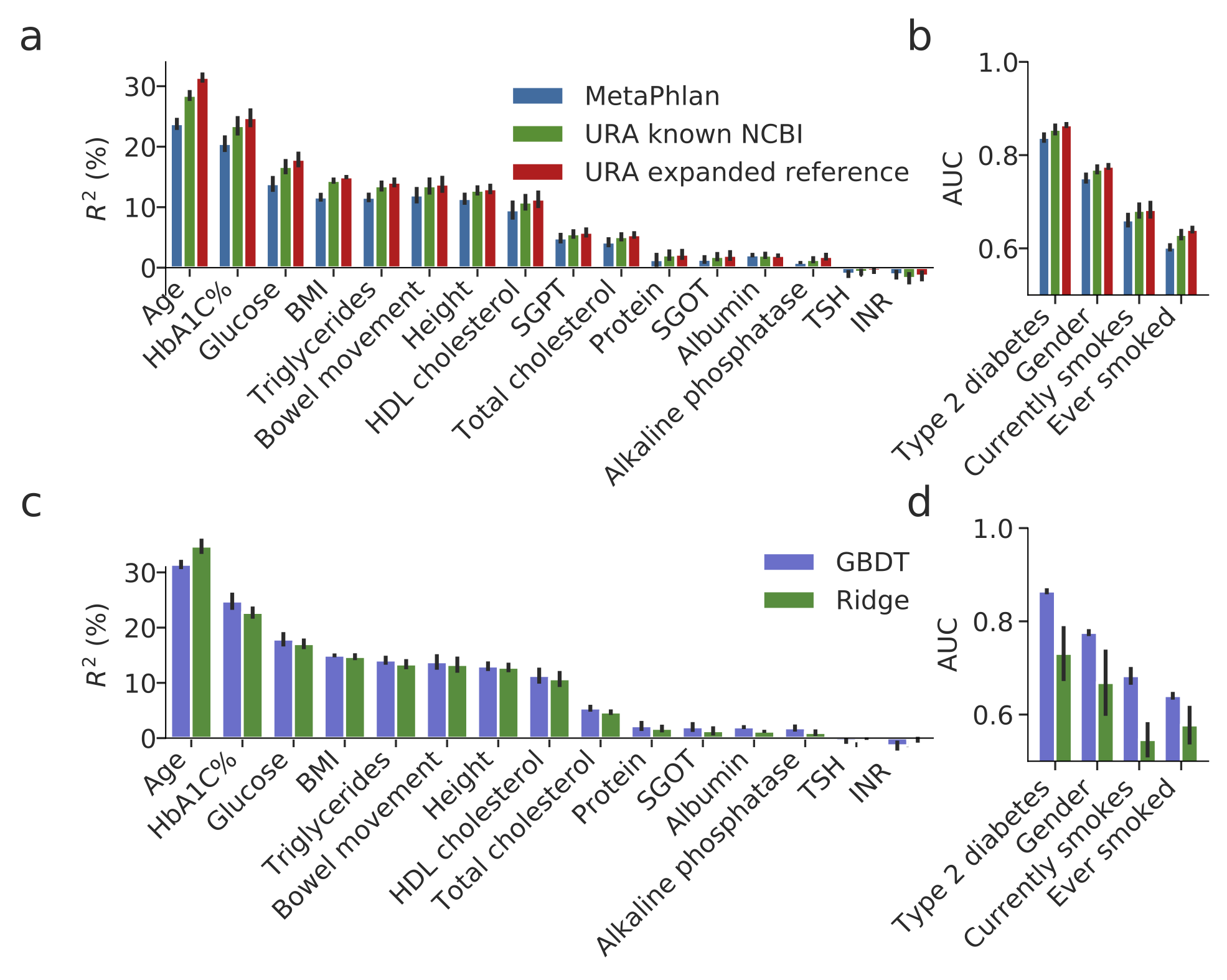

Supplement: S2 Fig — (a) Comparison of the predictive power of three different sets of species level abundance estimations of gut microbiome. In blue, predictions are performed using the baseline Metaphlan species abundances, in green predictions are performed on abundances calculated using the URA algorithm on the sub-set of SGBs that were known prior to the work of Pasolli et al. [30], and in red predictions are performed on URA abundances. For all three, the coefficient of determination (R2) of GBDT prediction of different phenotypes are obtained in a 10-fold cross validation scheme on the training set. (b) Same as (a), but shown is the area under the curve (AUC) for predicting binary phenotypes. (c) Comparison of the predictive power of GBDT model (in blue) versus Ridge regression model (in green). Coefficient of determination (R2) of prediction of different phenotypes based only on species level gut microbiome abundance. Results are obtained in a 10-fold cross validation scheme on the training set. (d) Same as (c), but shown is the area under the curve (AUC) for predicting binary phenotypes. (TIFF) [file pone.0265756.s002.tiff]

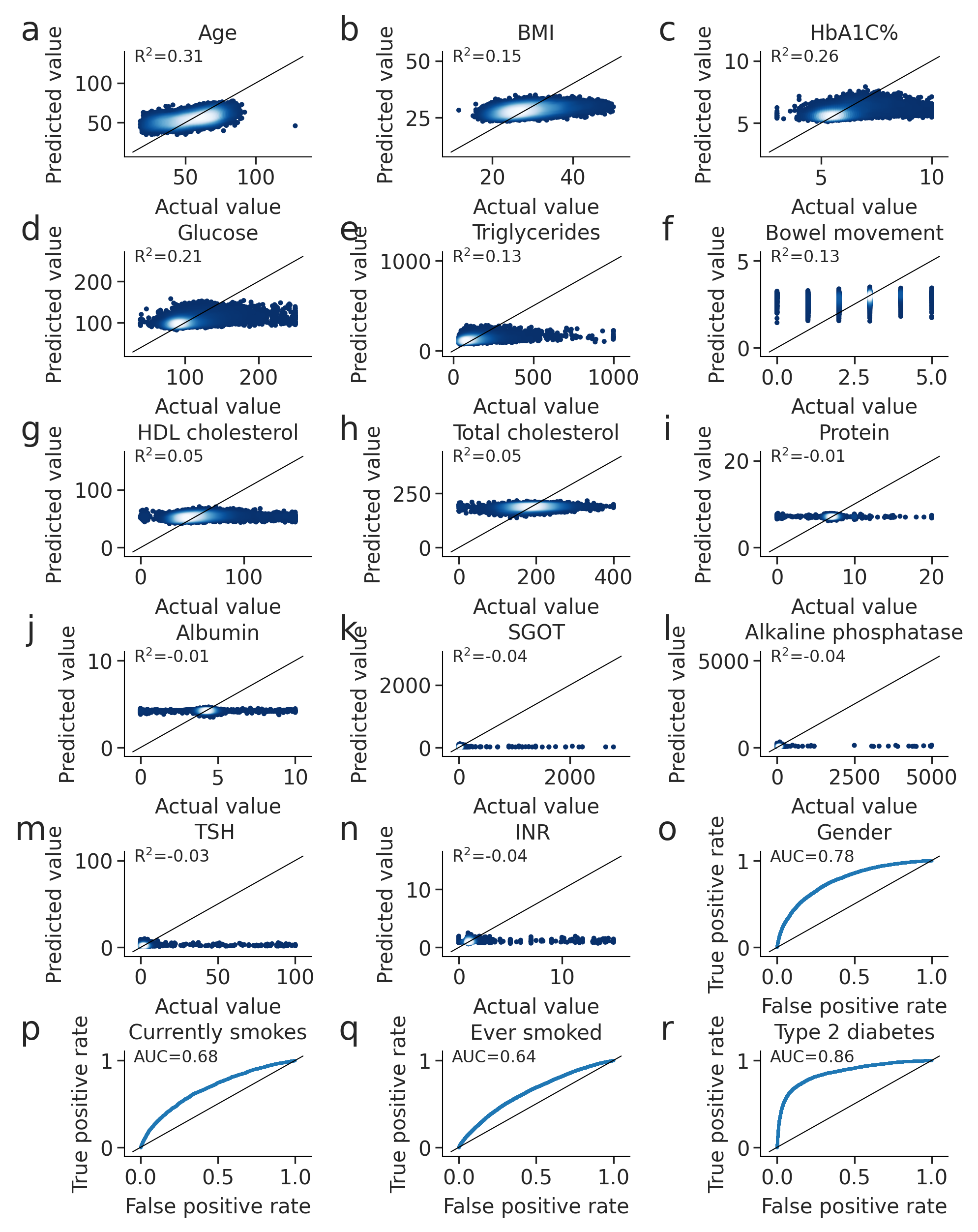

Supplement: S3 Fig — (a)-(n) Scatter plots of 10-fold cross-validation predicted values of quantitative phenotypes when training on the Israeli train cohort using GBDT. R2 of prediction is reported. Black line represents x = y. (o)-(r) ROC curve plots of 10-fold cross-validation predicted values of binary phenotypes when training on the Israeli train cohort using GBDT. AUC of prediction is reported. Black line represents x = y. (TIFF) [file pone.0265756.s003.tiff]

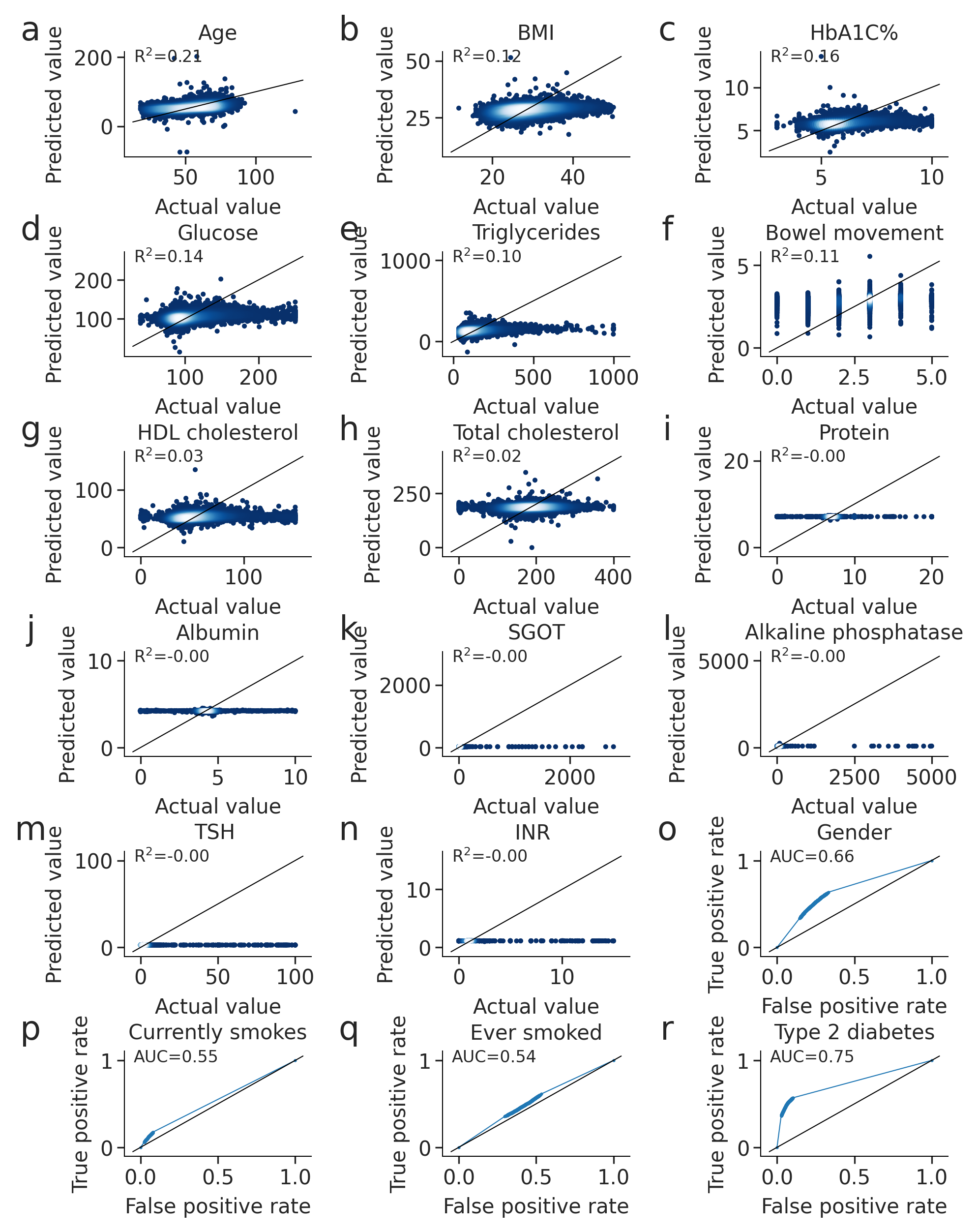

Supplement: S4 Fig — (a)-(n) Scatter plots of 10-fold cross-validation predicted values of quantitative phenotypes when training on the Israeli train cohort using Ridge regression. R2 of prediction is reported. Black line represents x = y. (o)-(r) ROC curve plots of 10-fold cross-validation predicted values of binary phenotypes when training on the Israeli train cohort using Ridge regression. AUC of prediction is reported. Black line represents x = y. (TIFF) [file pone.0265756.s004.tiff]

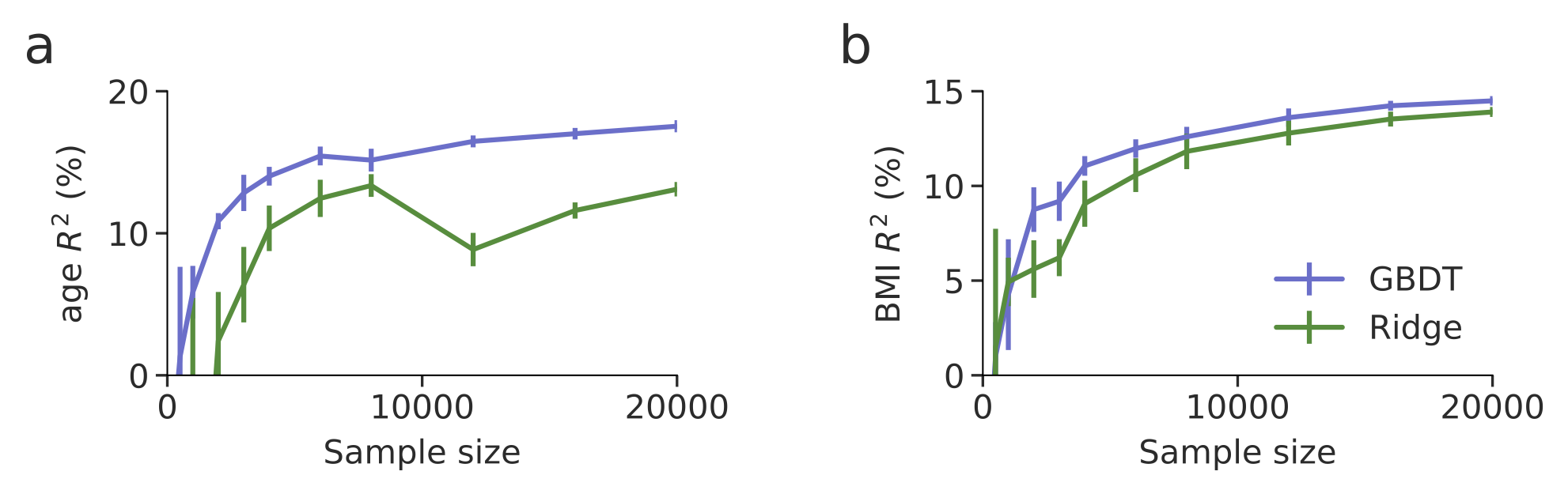

Supplement: S5 Fig — (a)—(b) Coefficient of determination (R2) and standard deviation error bars of predictions of age (a) and BMI (b) obtained using a GBDT (purple) or Ridge regression (green) models trained on sub-samples of the cohort train IL, of different sizes, and tested of the whole test US cohort. For each cohort size k, 10 random sub-samples of k individuals were obtained and the mean and standard deviation of their predictions are shown. (TIFF) [file pone.0265756.s005.tiff]
