# Supplementary material for: An atlas of robust microbiome associations with phenotypic traits based on large-scale cohorts from two continents
Source: PLoS One. 2022 Mar 24;17(3):e0265756. doi: 10.1371/journal.pone.0265756 (PMC8947124; doi:10.1371/journal.pone.0265756)
Supplement: S1 File — (DOCX) [file pone.0265756.s006.docx]

**Supplementary**

**Linear Model Predictions**

We compared two models; a linear model (with ridge regression regularization, **Supplementary**) and gradient boosted decision trees (GBDT) (**Methods**), using. only species RAs as input features to the model.

The linear models are attractive since their accuracy was almost similar to boosting decision trees and they are easier to interpret. However, boosting trees performed better across 11 of 12 phenotypes (age being the exception) that had significant predictions (overall mean R^2^ improvement of 0.02+/-0.011, **S2c, S2d Fig**) suggesting that non-additive interactions between different bacteria are predictive of several traits.

Our linear model obtained significant predictions for many traits (**S3, S4 Figs**) such as age (R^2^=0.21, for 10-fold cross validation on train IL samples), gender (AUC=0.64), HbA1C% (R^2^=0.15) and BMI (R^2^=0.12). We also found that prediction accuracy increases with cohort size, for the linear model as well (**Fig 4g-4i, S4 Fig**) and does not saturate even with a cohort of 1,000 individuals. Together, these results highlight the need for obtaining large cohorts for microbiome studies, as is known to be the case in the field of human genetics.

**Functional characterization of gut microbiome**

Most notably, the Lipopolysaccharide (LPS) biosynthesis pathway (ko00540) and the KDO2-lipid A biosynthesis module (M00060) were associated with adverse metabolic status (**Fig 6d, 6e**). Specifically, ko00540 and M00060 associated with increased levels of HbA1C% (P-values < 10^-3^ and 0.017 after FDR correction, respectively), BMI (P-values < 10^-3^ and 0.0014), fasting glucose (P-values < 10^-4^ and < 10^-2^) and fasting triglycerides (P-values < 10^-4^ and < 10^-3^) and with decreased levels of HDL-cholesterol (P-values < 10^-3^ and < 10^-2^). The LPS is a major component of the outer membrane of Gram-negative bacteria which is involved with toxicity, pathogenicity, antimicrobial resistance and other activities. LPS endotoxin purified from *E. coli* was previously shown to induce obese and insulin-resistant phenotypes when subcutaneously infused into mice [[1]](https://sciwheel.com/work/citation?ids=647076&pre=&suf=&sa=0). KDO2-lipid, the corresponding module, was shown to trigger defense-related responses by the human immune system to stimulate secretion of proinflammatory cytokines [[2]](https://sciwheel.com/work/citation?ids=647067&pre=&suf=&sa=0) .

Another biological pathway that was positively associated with HbA1C% (P-value = 0.04), fasting glucose (P-value = 0.039) & fasting triglycerides (P-value = 0.034) is the citrate cycle (ko00020), which is an energy production metabolic pathway found mainly in aerobic bacteria. In concordance with this pathway, we found two modules that are part of the citrate cycle pathway (M00009 & M00011) that also positively associated with HbA1C% (P-value = 0.017 for both modules) and fasting glucose (P-value = 0.042 and = 0.044). The finding that this metabolic pathway is in significant association with pro-disease markers may be related to an oxygenic environment in the gut. This is in concordance with previous studies showing that chronic inflammation with oxidative gut-environment causes an imbalance between obligate and facultative anaerobes and supports gut dysbiosis [[3–5]](https://sciwheel.com/work/citation?ids=3525942,4678277,7216176&pre=&pre=&pre=&suf=&suf=&suf=&sa=0,0,0).

We also found positive associations of vitamin metabolism modules, such as Menaquinone biosynthesis & Tetrahydrofolate biosynthesis (M00116 & M00126), with BMI (P-value = 0.019 and = 0.032, respectively), fasting triglycerides (P-value = 0.039 and = 0.04, respectively) and negative association with HDL-cholesterol (P-value = 0.026 and = 0.039), supporting previous findings of high concentration of menaquinone detected in the adipose tissues of obese adults [[6]](https://sciwheel.com/work/citation?ids=9825683&pre=&suf=&sa=0). Menaquinone produced by *Enterobacter cloacae* had been shown to have strong correlation to BMI [[7]](https://sciwheel.com/work/citation?ids=9825684&pre=&suf=&sa=0) and moreover, *E. cloacae* isolated from the gut of morbidly obese individuals induced obesity in germfree mice [[8]](https://sciwheel.com/work/citation?ids=172480&pre=&suf=&sa=0). Our analysis strengthens these links between bacteria producing Menaquinone and morbid obesity.

**References**

[1. Cani PD, Amar J, Iglesias MA, Poggi M, Knauf C, Bastelica D, et al. Metabolic endotoxemia initiates obesity and insulin resistance. Diabetes. 2007;56: 1761–1772. doi:10.2337/db06-1491](https://sciwheel.com/work/bibliography/647076)

[2. Creely SJ, McTernan PG, Kusminski CM, Fisher ff M, Da Silva NF, Khanolkar M, et al. Lipopolysaccharide activates an innate immune system response in human adipose tissue in obesity and type 2 diabetes. Am J Physiol Endocrinol Metab. 2007;292: E740-7. doi:10.1152/ajpendo.00302.2006](https://sciwheel.com/work/bibliography/647067)

[3. Rivera-Chávez F, Lopez CA, Bäumler AJ. Oxygen as a driver of gut dysbiosis. Free Radic Biol Med. 2017;105: 93–101. doi:10.1016/j.freeradbiomed.2016.09.022](https://sciwheel.com/work/bibliography/3525942)

[4. Henson MA, Phalak P. Microbiota dysbiosis in inflammatory bowel diseases: in silico investigation of the oxygen hypothesis. BMC Syst Biol. 2017;11: 145. doi:10.1186/s12918-017-0522-1](https://sciwheel.com/work/bibliography/4678277)

[5. Luca M, Di Mauro M, Di Mauro M, Luca A. Gut microbiota in alzheimer’s disease, depression, and type 2 diabetes mellitus: the role of oxidative stress. Oxid Med Cell Longev. 2019;2019: 4730539. doi:10.1155/2019/4730539](https://sciwheel.com/work/bibliography/7216176)

[6. Shea MK, Booth SL, Gundberg CM, Peterson JW, Waddell C, Dawson-Hughes B, et al. Adulthood obesity is positively associated with adipose tissue concentrations of vitamin K and inversely associated with circulating indicators of vitamin K status in men and women. J Nutr. 2010;140: 1029–1034. doi:10.3945/jn.109.118380](https://sciwheel.com/work/bibliography/9825683)

[7. Wang S, Li N, Li N, Zou H, Wu M. A comparative analysis of biosynthetic gene clusters in lean and obese humans. Biomed Res Int. 2019;2019: 6361320. doi:10.1155/2019/6361320](https://sciwheel.com/work/bibliography/9825684)

[8. Fei N, Zhao L. An opportunistic pathogen isolated from the gut of an obese human causes obesity in germfree mice. ISME J. 2013;7: 880–884. doi:10.1038/ismej.2012.153](https://sciwheel.com/work/bibliography/172480)
