# Supplementary material for: An atlas of robust microbiome associations with phenotypic traits based on large-scale cohorts from two continents
Source: PLoS One. 2022 Mar 24;17(3):e0265756. doi: 10.1371/journal.pone.0265756 (PMC8947124; doi:10.1371/journal.pone.0265756)
Supplement: S1 Dataset — (DOCX) [file pone.0265756.s008.docx]

**Minimal data set**

## 100 raw metagenomic samples from the Personal Nutrition Project [[1]](https://sciwheel.com/work/citation?ids=1005395&pre=&suf=&sa=0) on which the code can be run to validate the pipeline and reproduce the results obtained. These samples are available under bioproject PRJEB11532, with accession numbers detailed in supplementary (**S28 Table in S2 File**). The RAs of gut microbes of these samples, as calculated by the URA method, are given in supplementary (**S29 Table in S2 File**). These 100 samples could be made publicly available as they were obtained under a different academic informed consent that allows their sharing.

**Reference**

[1. Zeevi D, Korem T, Zmora N, Israeli D, Rothschild D, Weinberger A, et al. Personalized nutrition by prediction of glycemic responses. Cell. 2015;163: 1079–1094. doi:10.1016/j.cell.2015.11.001](https://sciwheel.com/work/bibliography/1005395)
